# 4-(Morpholin-4-yl)-3-(trifluoro­meth­yl)­benzonitrile

**DOI:** 10.1107/S1600536811020666

**Published:** 2011-06-11

**Authors:** Hoong-Kun Fun, Safra Izuani Jama Asik, Rajesha Kumar, Arun M. Isloor, K. N. Shivananda

**Affiliations:** a X-ray Crystallography Unit, School of Physics, Universiti Sains Malaysia, 11800 USM, Penang, Malaysia; b Organic Chemistry Division, Department of Chemistry, National Institute of Technology – Karnataka, Surathkal, Mangalore 575 025, India; c Department of Chemistry, Technion Israel Institute of Technology, Haifa 32000, Israel

## Abstract

In the title benzonitrile compound, C_12_H_11_F_3_N_2_O, an intra­molecular C—H⋯F hydrogen bond generates an *S*(7) ring motif. The trifluoro­methyl group is disordered over two orientations with a refined occupancy ratio of 0.549 (16):0.451 (16). The morpholine ring adopts a chair conformation. The benzene ring and mean plane of the morpholine ring make a dihedral angle of 58.04 (10)° with each other. In the crystal, mol­ecules are connected by inter­molecular C—H⋯F and C—H⋯O inter­actions to form *R* ^2^_2_(8) ring motifs. These inter­actions also link the mol­ecules into chains parallel to the [10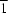] direction.

## Related literature

For general background and applications of materials related to the title compound, see: Raparti *et al.* (2009 ▶). For the synthesis of fluvoxamine, see: Schareina *et al.* (2004 ▶). For synthesis of the title compound, see: Kleemann *et al.* (2001 ▶). For graph-set theory, see: Bernstein *et al.* (1995 ▶). For bond-length data, see: Allen *et al.* (1987 ▶). For definition of puckering parameters, see: Cremer & Pople (1975 ▶).
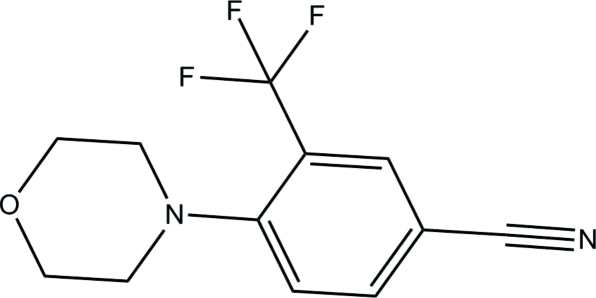


## Experimental

### 

#### Crystal data

C_12_H_11_F_3_N_2_O
*M* *_r_* = 256.23
Monoclinic, 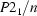

*a* = 12.7003 (12) Å
*b* = 6.8990 (7) Å
*c* = 13.3484 (13) Å
β = 91.668 (2)°
*V* = 1169.1 (2) Å^3^
*Z* = 4
Mo *K*α radiation
μ = 0.13 mm^−1^
*T* = 296 K
0.85 × 0.25 × 0.12 mm

#### Data collection

Bruker APEX DUO CCD area-detector diffractometer
Absorption correction: multi-scan (*SADABS*; Bruker, 2009 ▶) *T*_min_ = 0.899, *T*_max_ = 0.985
11929 measured reflections
3382 independent reflections
2399 reflections with *I* > 2σ(*I*)
*R*_int_ = 0.021

#### Refinement

*R*[*F* ^2^ > 2σ(*F* ^2^)] = 0.044
*wR*(*F* ^2^) = 0.132
*S* = 1.07
3382 reflections
192 parameters
H-atom parameters constrained
Δρ_max_ = 0.25 e Å^−3^
Δρ_min_ = −0.16 e Å^−3^

### 

Data collection: *APEX2* (Bruker, 2009 ▶); cell refinement: *SAINT* (Bruker, 2009 ▶); data reduction: *SAINT*; program(s) used to solve structure: *SHELXTL* (Sheldrick, 2008 ▶); program(s) used to refine structure: *SHELXTL*; molecular graphics: *SHELXTL*; software used to prepare material for publication: *SHELXTL* and *PLATON* (Spek, 2009 ▶).

## Supplementary Material

Crystal structure: contains datablock(s) global, I. DOI: 10.1107/S1600536811020666/rz2604sup1.cif

Structure factors: contains datablock(s) I. DOI: 10.1107/S1600536811020666/rz2604Isup2.hkl

Supplementary material file. DOI: 10.1107/S1600536811020666/rz2604Isup3.cml

Additional supplementary materials:  crystallographic information; 3D view; checkCIF report

## Figures and Tables

**Table 1:** Hydrogen-bond geometry (Å, °)

*D*—H⋯*A*	*D*—H	H⋯*A*	*D*⋯*A*	*D*—H⋯*A*
C2—H2*B*⋯F3^i^	0.97	2.49	3.242 (5)	135
C4—H4*A*⋯F1	0.97	2.23	2.909 (6)	126
C9—H9*A*⋯O1^ii^	0.93	2.47	3.3588 (16)	160

Symmetry codes: (i) 
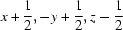
; (ii) 
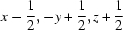
.

**Fig. 1.**
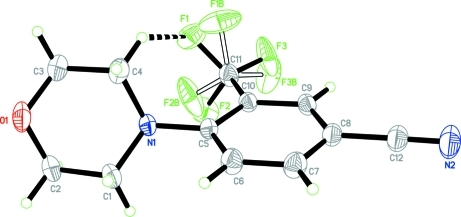
The structure of the title compound, showing 30% probability displacement ellipsoids.

**Fig. 2.**
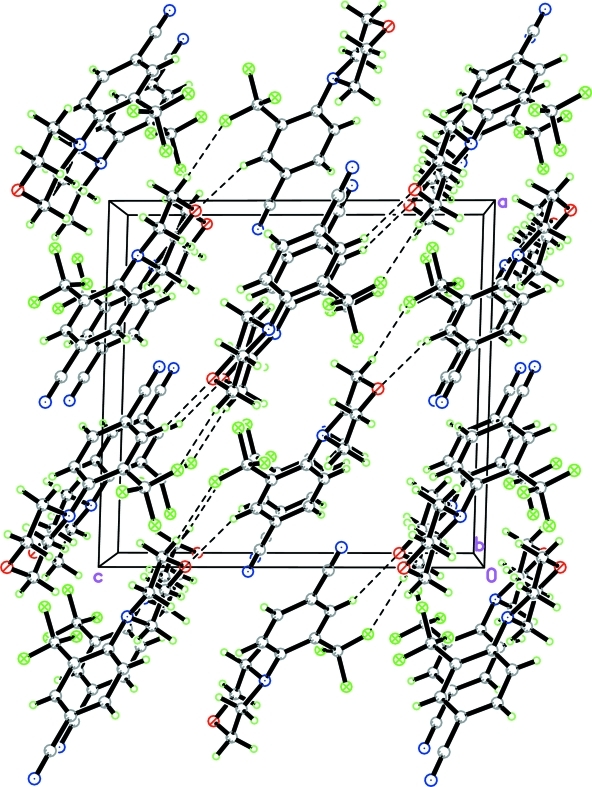
The crystal packing, viewed along the b axis, showing the chains parallel to the [1 0 1] direction. Hydrogen bonds are shown as dashed lines.

## supplementary crystallographic information

### Comment

Benzonitriles are of considerable interest in organic chemistry as an integral part of dyes, herbicides, agrochemicals, pharmaceuticals, and natural products. The nitrile group also serves as an important intermediate for a multitude of possible transformations into other functional groups and morpholine ring is important for antimicrobial activity (Raparti *et al.*, 2009). As an example, in the synthesis of *Fluvoxamine* (Schareina *et al.*, 2004), 4-(trifluoromethyl) benzonitrile, which is available from 4-chlorobenzotrifluoride by nickel-catalyzed cyanation on ton-scale, serves as an intermediate. Benzonitriles themselves are also of significant interest as substructures in biologically active agents. Bicalutamid and fadrozole are examples of pharmaceuticals containing an aromatic nitrile as part of the molecule. Prompted by these observations, we synthesized the title compound for studying its crystal structure.

In the title benzonitriles compound, an intramolecular C4—H4A···F1 hydrogen bond (Table 1) generates a seven-membered ring, producing an *S*(7) hydrogen bond ring motif (Fig. 1; Bernstein *et al.*, 1995). The trifluoromethyl group (F1–F3) is disordered over two orientations with refined occupancies of 0.549 (16) and 0.451 (16). The morpholine (C1–C4/N1/O1) ring adopts a chair conformation. The puckering parameters are Q = 0.5731 (18) Å, θ = 178.85 (17)°, φ = 322 (5)° (Cremer & Pople, 1975). The benzene ring (C6–C10) and  mean plane of the morpholine ring (C1–C4/O1/N1) make a dihedral angle of 58.04 (10)° with each other. The bond lengths (Allen *et al.*, 1987) and angles in the title of compound show the normal values.

In the crystal packing (Fig. 2), the molecules are connected by intermolecular interactions C2—H2B···F3 and C9—H9A···O1 hydrogen bonds to form *R*^2^_2_(8) ring motifs. These interactions also link the molecules into chains parallel to the [1 0 1] direction.

### Experimental

4-Fluoro-3-(trifluoromethyl)benzonitrile (3 g, 0.0158 mol) was taken in acetonitrile (50 ml) at 298–299 K under nitrogen atmosphere. Potassium carbonate (2.6 g, 0.019 mol) and morpholine (1.65 g, 0.019 mol) were added at the same temperature. The reaction mixture was heated to 353 K for 12 h. The reaction mixture was cooled to 298–299 K, concentrated under vacuum and the crude product was diluted with water (100 ml) and extracted with ethyl acetate (2x100 ml). The ethyl acetate layer was further washed with water (100 ml), brine solution, dried over Na_2_SO_4_ and concentrated to get the desired product as colourless crystalline solid, recrystallised from ethanol (Klemann *et al.*, 2001). Yield 3.8 g (94%), M.p.: 408–410 K.

### Refinement

Atoms F1, F2 and F3 are disordered over two sets of sites with a refined occupancy ratio of 0.549 (16):0.451 (16). All the H atoms were placed in calculated positions with C–H = 0.93 or 0.97 Å, The *U*_iso_ values were constrained to be 1.2*U*_eq_ of the carrier atoms.

### Crystal data

**Table:**

C_12_H_11_F_3_N_2_O	*F*(000) = 528
*M**_r_* = 256.23	*D*_x_ = 1.456 Mg m^−^^3^
Monoclinic, *P*2_1_/*n*	Mo *K*α radiation, λ = 0.71073 Å
Hall symbol: -P 2yn	Cell parameters from 3536 reflections
*a* = 12.7003 (12) Å	θ = 3.1–29.3°
*b* = 6.8990 (7) Å	µ = 0.13 mm^−^^1^
*c* = 13.3484 (13) Å	*T* = 296 K
β = 91.668 (2)°	Plate, colourless
*V* = 1169.1 (2) Å^3^	0.85 × 0.25 × 0.12 mm
*Z* = 4	

### Data collection

**Table:**

Bruker APEX DUO CCD area-detector diffractometer	3382 independent reflections
Radiation source: fine-focus sealed tube	2399 reflections with *I* > 2σ(*I*)
graphite	*R*_int_ = 0.021
φ and ω scans	θ_max_ = 30.0°, θ_min_ = 2.3°
Absorption correction: multi-scan (*SADABS*; Bruker, 2009)	*h* = −15→17
*T*_min_ = 0.899, *T*_max_ = 0.985	*k* = −9→9
11929 measured reflections	*l* = −18→18

### Refinement

**Table:**

Refinement on *F*^2^	Secondary atom site location: difference Fourier map
Least-squares matrix: full	Hydrogen site location: inferred from neighbouring sites
*R*[*F*^2^ > 2σ(*F*^2^)] = 0.044	H-atom parameters constrained
*wR*(*F*^2^) = 0.132	*w* = 1/[σ^2^(*F*_o_^2^) + (0.0566*P*)^2^ + 0.1831*P*] where *P* = (*F*_o_^2^ + 2*F*_c_^2^)/3
*S* = 1.07	(Δ/σ)_max_ = 0.001
3382 reflections	Δρ_max_ = 0.25 e Å^−^^3^
192 parameters	Δρ_min_ = −0.16 e Å^−^^3^
0 restraints	Extinction correction: *SHELXTL* (Sheldrick, 2008), Fc^*^=kFc[1+0.001xFc^2^λ^3^/sin(2θ)]^-1/4^
Primary atom site location: structure-invariant direct methods	Extinction coefficient: 0.010 (2)

### Special details

**Table:**

Geometry. All esds (except the esd in the dihedral angle between two l.s. planes) are estimated using the full covariance matrix. The cell esds are taken into account individually in the estimation of esds in distances, angles and torsion angles; correlations between esds in cell parameters are only used when they are defined by crystal symmetry. An approximate (isotropic) treatment of cell esds is used for estimating esds involving l.s. planes.
Refinement. Refinement of F^2^ against ALL reflections. The weighted R-factor wR and goodness of fit S are based on F^2^, conventional R-factors R are based on F, with F set to zero for negative F^2^. The threshold expression of F^2^ > 2sigma(F^2^) is used only for calculating R-factors(gt) etc. and is not relevant to the choice of reflections for refinement. R-factors based on F^2^ are statistically about twice as large as those based on F, and R- factors based on ALL data will be even larger.

### Fractional atomic coordinates and isotropic or equivalent isotropic displacement parameters (Å^2^)

**Table:**

	*x*	*y*	*z*	*U*_iso_*/*U*_eq_	Occ. (<1)
F1	0.7836 (5)	0.4513 (6)	0.0784 (4)	0.0736 (12)	0.549 (16)
F2	0.8776 (5)	0.2468 (9)	0.1535 (5)	0.0737 (13)	0.549 (16)
F3	0.7289 (6)	0.3126 (14)	0.2091 (5)	0.102 (2)	0.549 (16)
F1B	0.7282 (13)	0.4501 (8)	0.1030 (7)	0.110 (3)	0.451 (16)
F2B	0.8734 (6)	0.301 (2)	0.1241 (10)	0.107 (3)	0.451 (16)
F3B	0.7507 (7)	0.2605 (15)	0.2204 (4)	0.090 (2)	0.451 (16)
O1	0.98647 (10)	0.34879 (18)	−0.21269 (9)	0.0691 (4)	
N1	0.85532 (8)	0.17086 (16)	−0.07037 (8)	0.0412 (3)	
N2	0.45173 (11)	−0.3350 (2)	0.12877 (13)	0.0742 (4)	
C1	0.92779 (15)	0.0579 (2)	−0.12999 (16)	0.0747 (6)	
H1A	0.8918	0.0116	−0.1905	0.090*	
H1B	0.9527	−0.0536	−0.0919	0.090*	
C2	1.01990 (16)	0.1842 (3)	−0.15715 (18)	0.0829 (7)	
H2A	1.0570	0.2260	−0.0964	0.099*	
H2B	1.0685	0.1093	−0.1964	0.099*	
C3	0.91524 (13)	0.4581 (2)	−0.15705 (15)	0.0675 (5)	
H3A	0.8916	0.5687	−0.1965	0.081*	
H3B	0.9510	0.5067	−0.0970	0.081*	
C4	0.82075 (12)	0.3401 (2)	−0.12777 (13)	0.0598 (4)	
H4A	0.7746	0.4189	−0.0878	0.072*	
H4B	0.7816	0.2990	−0.1875	0.072*	
C5	0.77145 (9)	0.06434 (17)	−0.02829 (8)	0.0364 (3)	
C6	0.72846 (11)	−0.0982 (2)	−0.07585 (10)	0.0487 (3)	
H6A	0.7562	−0.1390	−0.1361	0.058*	
C7	0.64609 (11)	−0.2008 (2)	−0.03650 (11)	0.0505 (3)	
H7A	0.6187	−0.3087	−0.0699	0.061*	
C8	0.60446 (10)	−0.14135 (19)	0.05332 (10)	0.0418 (3)	
C9	0.64620 (9)	0.01808 (19)	0.10331 (9)	0.0394 (3)	
H9A	0.6182	0.0571	0.1637	0.047*	
C10	0.72972 (9)	0.12021 (17)	0.06375 (9)	0.0360 (3)	
C11	0.77589 (11)	0.2827 (2)	0.12573 (11)	0.0481 (3)	
C12	0.51905 (11)	−0.2485 (2)	0.09623 (11)	0.0517 (3)	

### Atomic displacement parameters (Å^2^)

**Table:**

	*U*^11^	*U*^22^	*U*^33^	*U*^12^	*U*^13^	*U*^23^
F1	0.100 (3)	0.0403 (11)	0.0802 (18)	−0.0085 (14)	−0.0012 (17)	−0.0078 (10)
F2	0.053 (2)	0.0799 (19)	0.086 (3)	−0.0015 (17)	−0.020 (2)	−0.0182 (16)
F3	0.085 (2)	0.134 (5)	0.090 (4)	−0.050 (2)	0.059 (2)	−0.073 (3)
F1B	0.175 (8)	0.0450 (17)	0.107 (4)	0.028 (3)	−0.034 (4)	−0.021 (2)
F2B	0.058 (3)	0.148 (7)	0.118 (6)	−0.054 (4)	0.036 (3)	−0.083 (5)
F3B	0.133 (5)	0.104 (4)	0.0318 (17)	−0.054 (3)	0.004 (2)	−0.013 (2)
O1	0.0764 (8)	0.0712 (7)	0.0617 (7)	−0.0107 (6)	0.0354 (6)	0.0108 (6)
N1	0.0425 (5)	0.0427 (5)	0.0392 (5)	−0.0004 (4)	0.0159 (4)	0.0028 (4)
N2	0.0601 (8)	0.0777 (10)	0.0852 (11)	−0.0232 (7)	0.0116 (7)	0.0158 (8)
C1	0.0833 (12)	0.0516 (9)	0.0924 (13)	0.0031 (8)	0.0588 (10)	0.0010 (8)
C2	0.0725 (11)	0.0692 (11)	0.1103 (16)	0.0081 (9)	0.0591 (11)	0.0188 (11)
C3	0.0664 (10)	0.0548 (9)	0.0829 (12)	−0.0047 (7)	0.0286 (9)	0.0168 (8)
C4	0.0516 (8)	0.0597 (9)	0.0687 (10)	−0.0008 (7)	0.0136 (7)	0.0229 (7)
C5	0.0377 (6)	0.0381 (6)	0.0337 (6)	−0.0008 (5)	0.0066 (4)	0.0022 (5)
C6	0.0578 (8)	0.0498 (7)	0.0391 (7)	−0.0086 (6)	0.0120 (6)	−0.0086 (5)
C7	0.0559 (8)	0.0448 (7)	0.0508 (8)	−0.0115 (6)	0.0041 (6)	−0.0066 (6)
C8	0.0362 (6)	0.0433 (6)	0.0461 (7)	−0.0038 (5)	0.0028 (5)	0.0078 (5)
C9	0.0361 (6)	0.0456 (6)	0.0368 (6)	0.0005 (5)	0.0074 (4)	0.0028 (5)
C10	0.0356 (6)	0.0380 (6)	0.0348 (6)	−0.0006 (4)	0.0058 (4)	−0.0012 (5)
C11	0.0506 (7)	0.0496 (7)	0.0447 (7)	−0.0063 (6)	0.0117 (6)	−0.0101 (6)
C12	0.0448 (7)	0.0528 (8)	0.0577 (8)	−0.0089 (6)	0.0026 (6)	0.0081 (6)

### Geometric parameters (Å, °)

**Table:**

F1—C11	1.328 (4)	C3—C4	1.511 (2)
F2—C11	1.357 (6)	C3—H3A	0.9700
F3—C11	1.294 (5)	C3—H3B	0.9700
F1B—C11	1.335 (5)	C4—H4A	0.9700
F2B—C11	1.245 (7)	C4—H4B	0.9700
F3B—C11	1.321 (6)	C5—C6	1.3925 (18)
O1—C3	1.4064 (19)	C5—C10	1.4060 (16)
O1—C2	1.415 (2)	C6—C7	1.3794 (18)
N1—C5	1.4230 (14)	C6—H6A	0.9300
N1—C4	1.4573 (18)	C7—C8	1.3863 (19)
N1—C1	1.4597 (17)	C7—H7A	0.9300
N2—C12	1.1388 (18)	C8—C9	1.3840 (18)
C1—C2	1.511 (2)	C8—C12	1.4449 (18)
C1—H1A	0.9700	C9—C10	1.3902 (16)
C1—H1B	0.9700	C9—H9A	0.9300
C2—H2A	0.9700	C10—C11	1.5023 (18)
C2—H2B	0.9700		
			
C3—O1—C2	109.97 (12)	C5—C6—H6A	119.0
C5—N1—C4	113.82 (10)	C6—C7—C8	119.37 (12)
C5—N1—C1	115.51 (11)	C6—C7—H7A	120.3
C4—N1—C1	109.04 (12)	C8—C7—H7A	120.3
N1—C1—C2	109.13 (14)	C9—C8—C7	120.10 (11)
N1—C1—H1A	109.9	C9—C8—C12	119.81 (12)
C2—C1—H1A	109.9	C7—C8—C12	120.08 (12)
N1—C1—H1B	109.9	C8—C9—C10	120.36 (11)
C2—C1—H1B	109.9	C8—C9—H9A	119.8
H1A—C1—H1B	108.3	C10—C9—H9A	119.8
O1—C2—C1	111.47 (17)	C9—C10—C5	120.27 (11)
O1—C2—H2A	109.3	C9—C10—C11	117.32 (11)
C1—C2—H2A	109.3	C5—C10—C11	122.33 (11)
O1—C2—H2B	109.3	F2B—C11—F3	118.8 (5)
C1—C2—H2B	109.3	F2B—C11—F3B	107.3 (6)
H2A—C2—H2B	108.0	F2B—C11—F1	79.4 (6)
O1—C3—C4	112.06 (14)	F3—C11—F1	108.2 (4)
O1—C3—H3A	109.2	F3B—C11—F1	125.4 (4)
C4—C3—H3A	109.2	F2B—C11—F1B	110.7 (4)
O1—C3—H3B	109.2	F3—C11—F1B	80.8 (4)
C4—C3—H3B	109.2	F3B—C11—F1B	101.4 (5)
H3A—C3—H3B	107.9	F3—C11—F2	104.7 (4)
N1—C4—C3	109.77 (13)	F3B—C11—F2	88.5 (5)
N1—C4—H4A	109.7	F1—C11—F2	101.9 (3)
C3—C4—H4A	109.7	F1B—C11—F2	129.9 (6)
N1—C4—H4B	109.7	F2B—C11—C10	116.0 (4)
C3—C4—H4B	109.7	F3—C11—C10	114.2 (3)
H4A—C4—H4B	108.2	F3B—C11—C10	109.8 (4)
C6—C5—C10	117.81 (11)	F1—C11—C10	115.1 (2)
C6—C5—N1	121.60 (11)	F1B—C11—C10	110.7 (3)
C10—C5—N1	120.59 (11)	F2—C11—C10	111.6 (3)
C7—C6—C5	122.07 (12)	N2—C12—C8	178.91 (18)
C7—C6—H6A	119.0		
			
C5—N1—C1—C2	−172.23 (15)	C8—C9—C10—C5	1.04 (18)
C4—N1—C1—C2	58.1 (2)	C8—C9—C10—C11	−175.72 (12)
C3—O1—C2—C1	58.5 (2)	C6—C5—C10—C9	−1.85 (18)
N1—C1—C2—O1	−59.4 (2)	N1—C5—C10—C9	178.54 (11)
C2—O1—C3—C4	−57.5 (2)	C6—C5—C10—C11	174.75 (12)
C5—N1—C4—C3	172.24 (13)	N1—C5—C10—C11	−4.86 (18)
C1—N1—C4—C3	−57.19 (18)	C9—C10—C11—F2B	141.6 (9)
O1—C3—C4—N1	57.6 (2)	C5—C10—C11—F2B	−35.0 (9)
C4—N1—C5—C6	96.34 (15)	C9—C10—C11—F3	−2.1 (6)
C1—N1—C5—C6	−30.95 (19)	C5—C10—C11—F3	−178.8 (5)
C4—N1—C5—C10	−84.08 (15)	C9—C10—C11—F3B	19.9 (5)
C1—N1—C5—C10	148.64 (14)	C5—C10—C11—F3B	−156.8 (5)
C10—C5—C6—C7	1.4 (2)	C9—C10—C11—F1	−128.2 (4)
N1—C5—C6—C7	−178.98 (13)	C5—C10—C11—F1	55.1 (4)
C5—C6—C7—C8	−0.1 (2)	C9—C10—C11—F1B	−91.2 (8)
C6—C7—C8—C9	−0.7 (2)	C5—C10—C11—F1B	92.2 (8)
C6—C7—C8—C12	−179.22 (13)	C9—C10—C11—F2	116.3 (3)
C7—C8—C9—C10	0.27 (19)	C5—C10—C11—F2	−60.4 (3)
C12—C8—C9—C10	178.77 (12)		

### Hydrogen-bond geometry (Å, °)

**Table:**

*D*—H···*A*	*D*—H	H···*A*	*D*···*A*	*D*—H···*A*
C2—H2B···F3^i^	0.97	2.49	3.242 (5)	135.
C4—H4A···F1	0.97	2.23	2.909 (6)	126.
C9—H9A···O1^ii^	0.93	2.47	3.3588 (16)	160.

Symmetry codes: (i) *x*+1/2, −*y*+1/2, *z*−1/2; (ii) *x*−1/2, −*y*+1/2, *z*+1/2.
